# Ibuprofen results in alterations of human fetal testis development

**DOI:** 10.1038/srep44184

**Published:** 2017-03-10

**Authors:** Millissia Ben Maamar, Laurianne Lesné, Kristin Hennig, Christèle Desdoits-Lethimonier, Karen R. Kilcoyne, Isabelle Coiffec, Antoine D. Rolland, Cécile Chevrier, David M. Kristensen, Vincent Lavoué, Jean-Philippe Antignac, Bruno Le Bizec, Nathalie Dejucq-Rainsford, Rod T. Mitchell, Séverine Mazaud-Guittot, Bernard Jégou

**Affiliations:** 1 Institut national de la santé et de la recherche médicale (Inserm), Institut de recherche en santé, environnement et travail (Irset – Inserm UMR 1085), Université de Rennes 1, 9 Avenue Léon Bernard, F-35000 RENNES, France; 2 LUNAM Université, Oniris, USC INRA 1329, Laboratoire d’Etude des Résidus et Contaminants dans les Aliments (LABERCA), Nantes, F-44307, France; 3 MRC Centre for Reproductive Health, University of Edinburgh, Queens Medical Research Institute, 47 Little France Crescent, Edinburgh, EH16 4TJ, UK; 4 Laboratorium of Genomic and Molecular Biomedicine, Department of Biology, University of Copenhagen, Ole Maaløes Vej 5, DK-2200 Copenhagen N, Denmark; 5 CHU de Rennes, Service de Gynécologie, Hôpital Sud, 16, boulevard de Bulgarie, F-35700 Rennes, France; 6 Ecole des hautes études en santé publique (EHESP), Avenue Léon Bernard, F-35043 RENNES, France

## Abstract

Among pregnant women ibuprofen is one of the most frequently used pharmaceutical compounds with up to 28% reporting use. Regardless of this, it remains unknown whether ibuprofen could act as an endocrine disruptor as reported for fellow analgesics paracetamol and aspirin. To investigate this, we exposed human fetal testes (7–17 gestational weeks (GW)) to ibuprofen using *ex vivo* culture and xenograft systems. Ibuprofen suppressed testosterone and Leydig cell hormone INSL3 during culture of 8–9 GW fetal testes with concomitant reduction in expression of the steroidogenic enzymes *CYP11A1, CYP17A1* and *HSD17B3*, and of *INSL3*. Testosterone was not suppressed in testes from fetuses younger than 8 GW, older than 10–12 GW, or in second trimester xenografted testes (14–17 GW). *Ex vivo*, ibuprofen also affected Sertoli cell by suppressing AMH production and mRNA expression of *AMH, SOX9, DHH,* and *COL2A1*. While PGE2 production was suppressed by ibuprofen, PGD2 production was not. Germ cell transcripts *POU5F1, TFAP2C, LIN28A, ALPP* and *KIT* were also reduced by ibuprofen. We conclude that, at concentrations relevant to human exposure and within a particular narrow ‘early window’ of sensitivity within first trimester, ibuprofen causes direct endocrine disturbances in the human fetal testis and alteration of the germ cell biology.

Analgesics, including paracetamol and non-steroidal anti-inflammatory drugs (NSAIDs) such as aspirin and ibuprofen, are among the most widely used and environmentally prevalent pharmaceutical drugs in the world^1^. They are regarded as extremely effective medications and are widely used for self-medication, including by pregnant women during early pregnancy^2^,^3^,^4^. During pregnancy, analgesics are generally taken to relieve migraine, pain, and fever, but are also used in inflammatory conditions and are frequently used during preterm labor^1^,^5^,^6^,^7^,^8^. These medications are known to cross the placenta and to be present in meconium, neonatal urine, and in breast milk, indicating painkiller transmission from the mother to the fetus and to neonates^9^,^10^,^11^,^12^,^13^,^14^,^15^. Attention has recently been focused on the associations between analgesic use during the second and third trimesters of pregnancy and a number of unwanted effects in children. These include low birthweight, risk of premature closure of the ductus arteriosus, cardiac defects, decreased fetal and neonatal renal function, and asthma^16^,^17^,^18^,^19^,^20^. Concerns have also been raised recently about the use of over-the-counter painkillers during the first and second trimesters of pregnancy and an association with congenital cryptorchidism, the most frequent congenital reproductive disorder in newborn boys^7^,^8^,^21^,^22^, as well as with shorter anogenital distance (AGD) in male infants^23^,^24^, the latter being a biomarker for androgen action during fetal life. A series of studies undertaken *in utero* in the rat and *ex vivo* in rat and human fetal testes and in xenografted mouse models have shown that paracetamol and the NSAIDs aspirin and indomethacin can disrupt the testicular endocrine system. These disruptive endocrine effects may highlight analgesic involvement in fetal maldescended testes^1^,^7^,^25^,^26^,^27^,^28^.

Ibuprofen is the only of the 3 most common over-the-counter painkiller with paracetamol and aspirin, whose endocrine disruptive potential has not yet been investigated *ex vivo*. This is despite the fact that its consumption has continued to increase even though aspirin consumption has decreased^1^,^6^. The percentage of pregnant women reporting ibuprofen use averages 10%, but this varies widely across studies, ranging from 0.5% to over 28%^3^,^6^,^7^,^16^,^29^,^30^,^31^,^32^,^33^,^34^. In addition to cryptorchidism^7^, ibuprofen use by pregnant women has also been associated with hypospadias^35^, another congenital abnormality featuring a midline fusion defect of the male ventral urethra. The latter association was not found in another study^36^. Cryptorchidism and hypospadias are associated disorders and although some cases arise from genetic syndromes, most cases remain idiopathic^37^. However, both cryptorchidism and hypospadias most probably reflect subnormal levels of androgens during the development of the male urogenital tract^38^,^39^.

In this study, we investigated whether ibuprofen disrupts the endocrine system and the differentiation of the human fetal testis during the first and second trimesters of pregnancy. We used a combination of an organotypic culture system (Fetal Gonad Assay; FEGA) based on culturing human fetal testes fragments, and a human fetal testes xenograft system. These approaches, have been separately used for the study of endocrine-disrupting substances^26^,^27^,^40^,^41^, but never integrated in the same series of experiments. In the FEGA system, after 1 to 3 days of exposure to ibuprofen at 10^−7^–10^−4^ M, we assessed the gross morphology, endocrine function, and gene expression for the main cell types of the first trimester human fetal testis *ex vivo*, whilst the xenograft system was used to determine the effect of prolonged (7 day) ibuprofen exposure on the endocrine function of the second trimester testis.

## Results

### Ibuprofen and Leydig cell morphology and function

**Figure 1:**
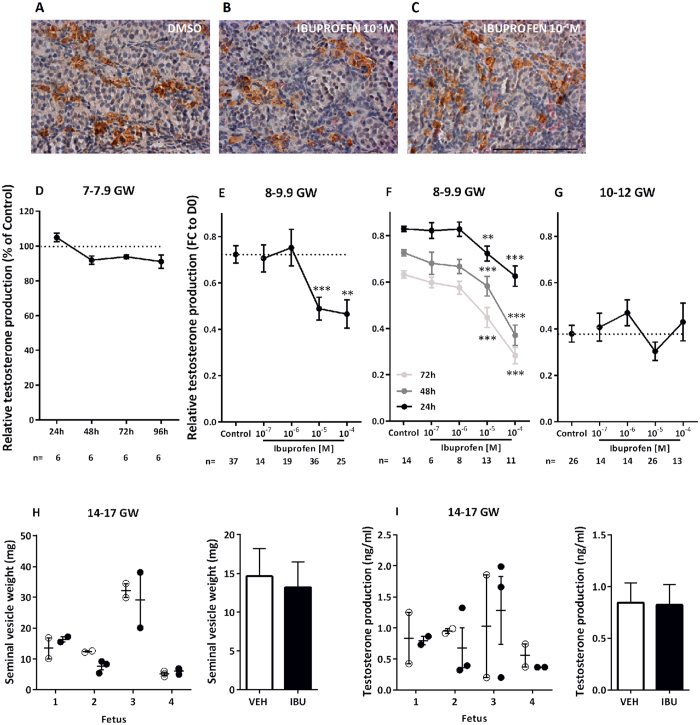
Ibuprofen and Leydig cell steroidogenic function. (**A–C**) Representative images of CYP11A1 immunostaining in explants of a 7.9 gestational week (GW) human fetal testis cultured with DMSO or ibuprofen. CYP11A1 appears brown (3,3′-diaminobenzidine tetrahydrochloride staining), and sections were counterstained with hematoxylin. Scale bar = 100 μm. (**D**) Testosterone production as a function of culture duration in 7–7.9 GW human fetal testes in the presence of DMSO (Control) or 10^−5 ^M of ibuprofen. Results are expressed as fold change from the respective control testis (% of Control). Values are means ± SEM of 6 testes from 6 different fetuses. Repeated measures analysis of variance (ANOVA) on ranks was performed (*p* = 0.172). (**E–G**) Testosterone production after culture of 8-9.9 GW (**E**,**F**) and 10–12 GW human fetal testes (**G**) in the presence of DMSO (Control) or 10^−7^–10^−4 ^M of ibuprofen for 72 h (**E**,**G**) and for 24, 48 and 72 h (**F**). Results are expressed as fold change from the first day of culture (FC to D0). Values are means ± SEM of 14–37 testes from 14–37 fetuses for the 8–9.9 GW group, 6–14 testes from 6–14 fetuses for the 8–9.9 GW at 24, 48 and 72 h group, and 13–21 testes from 13–21 fetuses for 10–12 GW. ANOVAs with a random fetus effect were performed using unstructured covariance matrices. A Wilcoxon test was also performed for pairwise comparisons (**p < 0.01, ***p < 0.001). (**H**) Seminal vesicle weight and (**I**) plasma testosterone production in host mice carrying human fetal testis xenografts (14–17 GW; n = 4 fetuses) after 7 days (7d) of exposure to vehicle (Corn Oil; open circles) or ibuprofen (10 mg/kg 3 times daily; closed circles) with overall mean ± SEM for vehicle (white bars) and ibuprofen (black bars). Values are means ± SEM from 4 fetuses. Data analyzed by two-way ANOVA.

No apparent changes in general morphology or Leydig cell marker expression was seen in the ibuprofen-exposed FEGA explants as compared to the non-exposed (Fig. 1A–C).

In the 7–7.9 gestational week (GW) 10^−5 ^M ibuprofen-treated testes, no significant effect was observed on testosterone levels when compared to the non-treated testes (Fig. 1D). By contrast, when the 8–9.9 GW testes were exposed *ex vivo* to ibuprofen, a significant dose-dependent testosterone decrease was seen after 72 h of exposure (slope β = −0.076, *p* < 0.0001); the pairwise comparisons versus the control condition evidenced an effect with 10^−5 ^M (−32.3%; *p* < 0.001 and with 10^−4 ^M (−35.6%; *p* < 0.01) (Fig. 1E). In the 8–9.9 GW testes, we also observed time- and dose-dependent effects of ibuprofen (after 24 h, slope β = −0.052, *p* < 0.0001; after 48 h, slope β = −0.081, *p* < 0.0001; after 72 h, slope β = −0.085, *p* < 0.0001), with the decrease of testosterone occurring after only 24 h of exposure: −12.8% with 10^−5 ^M (*p* < 0.01); and −24.6% with 10^−4 ^M (*p* < 0.001). This dose-dependent decrease was amplified after 48 h (−19.8 and −49%, respectively; both *p* < 0.001), and further amplified after 72 h (−29.3% and −55.3%, respectively; both *p* < 0.001) (Fig. 1F). In contrast, no effect was observed *ex vivo* on testosterone levels in the 10–12 GW testes for doses of ibuprofen of 10^−7^–10^−4^ M (Fig. 1G).

Given the apparent specific time window for testosterone reduction during the mid-late first trimester, we then investigated the effect of ibuprofen exposure during the second trimester (14–17 GW) using the xenograft system. Exposure to a 7 day therapeutic regimen of ibuprofen (10 mg/kg, 3 times daily) did not affect testosterone production from the human fetal testis as determined by both host mouse seminal vesicle weight (14.63 mg *versus* 13.21 mg; p = 0.62; n = 4 Fig. 1H) and plasma testosterone (0.84 *versus* 0.82 ng/ml; p = 0.85; n = 4; Fig. 1I) in host mice. Similarly, plasma 5-dihydrotestosterone (5α-DHT) was unaffected by exposure to ibuprofen (3.26 *versus* 2.44 ng/ml; p = 0.56; n = 4; Fig. S1). Mean plasma ibuprofen concentration 1 hour after the final dose in ibuprofen-exposed host mice was 2.78 ± 0.55 μg/ml and undetectable in vehicle-exposed controls (Fig. S1)

**Figure 2:**
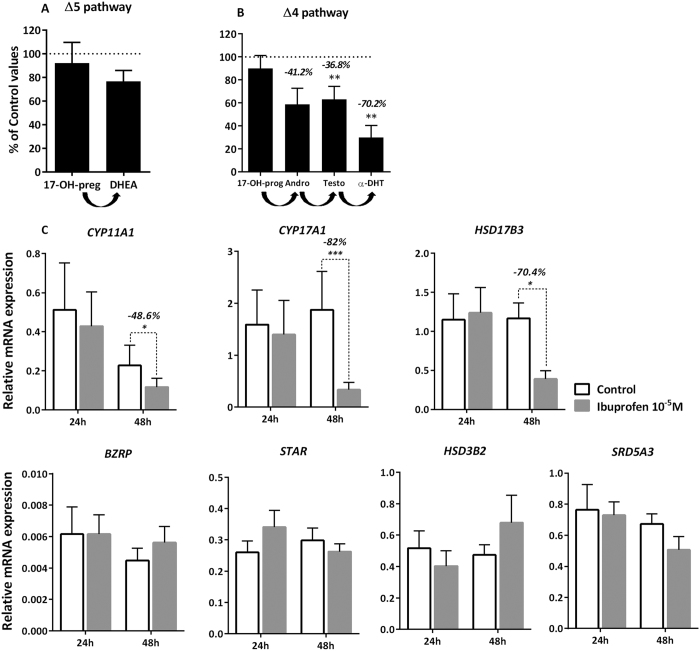
Ibuprofen and global steroidogenesis. (**A**,**B**) Global analysis of ibuprofen effects on the Δ5 (**A**) and Δ4 pathways (**B**) of testosterone production using GC-MS/MS. Human fetal testis explants (8–9.9 GW) were incubated for 48 h with DMSO (Control) or 10^−5 ^M of ibuprofen. Steroid precursors of the Δ5 and Δ4 pathways were measured by GC-MS/MS in the media. Values are mean ± SEM of 8 fetuses pooled in 5 independent experiments, and are expressed as the percentage of variation from the control. **p < 0.01 by non-parametric signed rank Wilcoxon test on paired data. (**C**) Quantitative RT-PCR of *BZRP, STAR, CYP11A1, CYP17A1, HSD17B3, HSD3B2* and *SRD5A3* was performed on control testes (white bars) and testes treated with 10^−5 ^M of ibuprofen (grey bars) for 24 and 48 h. Each column shows a pool of 11–15 fetal testes. Each bar represents the mean ± SEM of the fold change in target gene expression relative to the reference gene *RPLP0*. A non-parametric signed rank Wilcoxon test on paired data was performed (*p < 0.05, **p < 0.01, ***p < 0.001).

Using gas chromatography-tandem mass spectrometry (GC/MS-MS), we then measured the entire complement of detectable steroids both in the FEGA testis explants themselves and in the media after 48 h of exposure to 10^−5 ^M of ibuprofen in the 8–9.9 GW testes (Fig. 2A,B). Our results reveal that in this fetal age-range, only 3 steroids were found to be above the threshold of the GC/MS-MS technique in the non-treated fetal testis: pregnenolone (1.39 ± 0.7 ng/mg of testicular tissue); dehydroepiandrosterone (DHEA) (1.7 ± 1.1 ng/mg of testicular tissue); and testosterone (54.4 ± 25.7 ng/mg of testicular tissue). Levels of these 3 steroids in the explants were not significantly affected by ibuprofen at 10^−5 ^M (data not shown). Parallel investigation in the corresponding culture media allowed us to detect, for the first time in humans, 6 steroids in the control (*i.e*. unexposed) condition in both the Δ5 and Δ4 steroid pathways: - Δ5, 17-hydroxy-pregnenolone (17-OH-preg) (4.3 ± 1.9 ng/mL); DHEA (21.3 ± 4.7 ng/mL) (Fig. 2A); - Δ4, 17-hydroxy-progesterone (17-OH-prog) (9.6 ± 5.9 ng/mL); androstenedione (183.7 ± 97.0 ng/mL); testosterone (1597.8 ± 722.5 ng/mL); and 5-dihydrotestosterone (5α-DHT) (7.1 ± 8.0 ng/mL of culture media) (Fig. 2B). Of these, the potent androgens testosterone and α-DHT were found to be significantly inhibited by 10^−5 ^M of ibuprofen (36.8 and 70.2%, respectively at *p* < 0.01). The levels of the other androgen androstenedione were also reduced (−41.2%), but this was not statistically significant at *p* < 0.05 (Fig. 2B).

After 48 h of exposure to ibuprofen, the expression of 3 genes that encode for steroidogenic enzymes was markedly decreased: by 48.6% (*p* < 0.05) for *CYP11A1,* by 82% (*p* < 0.001) for *CYP17A1,* and by 70.4% (*p* < 0.05) for *HSD17B3* (Fig. 2C). By contrast, the mRNA levels of *HSD3B2* and *SRD5A3,* 2 other genes which encode enzymes of the steroidogenic pathway, as well as the mRNA levels of *BZRP* and *STAR,* the 2 genes encoding proteins involved in cholesterol transport to the mitochondria, were not affected by exposure to ibuprofen (Fig. 2C).

**Figure 3:**
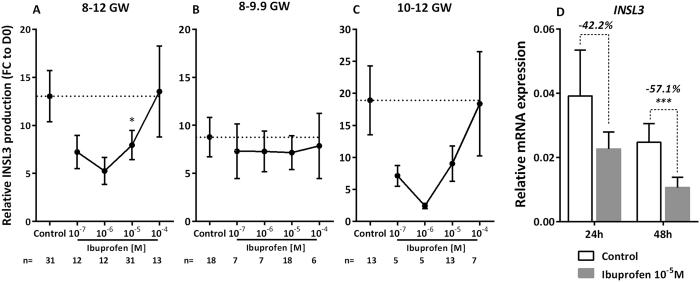
Ibuprofen and production of INSL3 by Leydig cells. (**A**–**C)** Ibuprofen and INSL3 production after exposure to ibuprofen during 72 h of human fetal testis culture at (**A**) 8–12 gestational weeks (GW), (**B**) 8–9.9 GW, and (**C**) 10–12 GW. Results are expressed as fold change from the first day of culture (FC to D0). Values are means ± SEM (*n* = 12–31 testes from 12–31 fetuses). ANOVAs with a random fetus effect were performed using unstructured covariance matrices. A Wilcoxon test was performed for pairwise comparisons. (*p < 0.05). (**D**) Quantitative RT-PCR for *INSL3* was performed on control testes (white bars) and testes treated with 10^−5 ^M of ibuprofen (grey bars) for 24 and 48 h. Each column is made up of a pool of 11–15 fetal testes. Each bar represents the mean ± SEM of the fold change in target gene expression relative to *RPLP0* reference gene. A non-parametric signed rank Wilcoxon test on paired data was performed (***p < 0.001).

The other key fetal Leydig cell-produced hormone investigated was INSL3. For explants from 8–12 week gestation fetuses, INSL3 levels were dose-dependently and significantly decreased after 72 h of exposure to ibuprofen at doses ranging from 10^−7^–10^−4 ^M, (slope β = −0.155, *p* = 0.007) (Fig. 3A). At 10^−5 ^M the decrease of INSL3 (−39%) was significant (*p* < 0.05), but at 10^−4 ^M no significant difference was observed. To assess whether a window of sensitivity exists, we again divided the fetuses into 2 age groups, 8–9.9 GW and 10–12 GW. In fact, there was a significant dose-response decrease in INSL3 levels for the 10–12 GW age group (slope β = −0.181, *p* = 0.03) (Fig. 3B,C). In addition to decreased INSL3 production, after 48 h of culture with 10^−5 ^M of ibuprofen, *INSL3* transcripts were also repressed by 57.1% (*p* < 0.001) (Fig. 3D). As in previous studies^26^,^42^, INSL3 production appears to have substantial interindividual variability.

### Ibuprofen and Sertoli cell morphology and function

**Figure 4:**
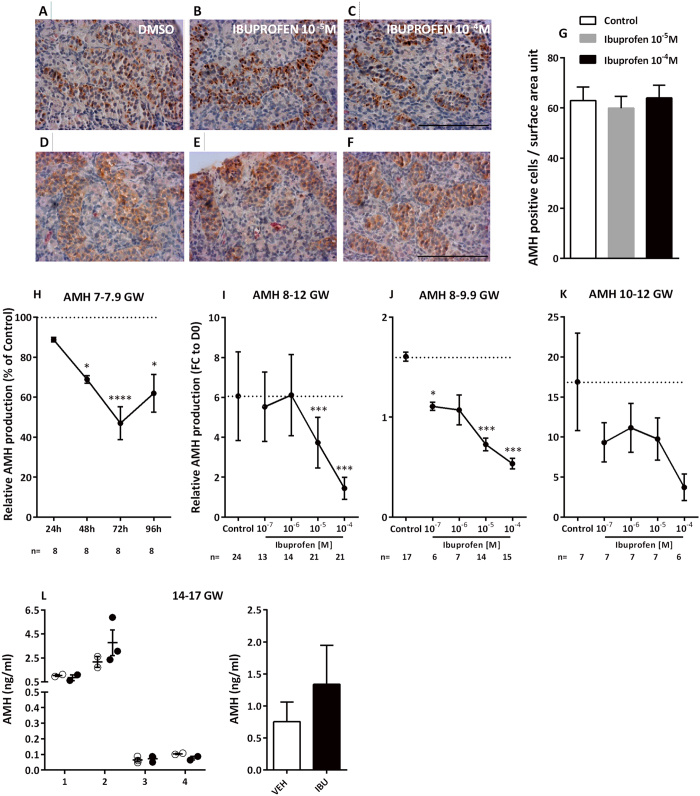
Ibuprofen and Sertoli cell function. (**A**–**F**) Representative images of KRT18 (**A**–**C**) and AMH (**D**–**F**) immunostaining in cultured explants of 7–7.9-gestational week (GW) human fetal testis. KRT18 and AMH appear brown (3,3-diaminobenzidine tetrahydrochloride staining), and sections were counterstained with hematoxylin. Scale bar = 100 μm. (**G**) Sertoli cell numbers were determined by counting AMH-positive cells in control testes (white bars) and testes treated with 10^−5 ^M (grey bars) and 10^−4 ^M (black bars) of ibuprofen for 72 h. Data are presented as the number of cells per surface area unit (0.01 mm^2^) (means ± SEM) based on 1 or 2 explants per treatment in 7 fetuses. A Wilcoxon test was performed for pairwise comparisons. (**H**) AMH production after 24, 48, and 72 h of exposure to DMSO (Control) or 10^−5 ^M of ibuprofen in 7–7.9 GW human fetal testes. Results are expressed as fold changes from the control testis (% of Ctrl). Values are means ± SEM for 7 testes from 7 fetuses. Repeated measures analysis of variance (ANOVA) on ranks was performed (*p < 0.05, ****p < 0.0001). (**I–K**) AMH production after culture of 8–12 GW (**I**); 8–9.9 GW (**J**); and 10–12 GW (**K**) human fetal testes in the presence of the control solvent DMSO (Control) or 10^−7^–10^−4 ^M of ibuprofen. Results are expressed as fold change from the first day of culture (FC to D0). Values are means ± SEM of 6–17 testes from 6–17 fetuses for the 8–9.9 GW, and of 6–7 testes from 6–7 fetuses for the 10–12 GW. ANOVAs with a random fetus effect were performed using unstructured covariance matrices. A Wilcoxon test was performed for pairwise comparisons (*p < 0.05; ***p < 0.001). (**L**) Plasma AMH in individual host mice carrying human fetal testis xenografts (14–17 GW; n = 4 fetuses) after 7d exposure to vehicle (Corn Oil; open circles) or ibuprofen (10 mg/kg 3 times daily; closed circles) with overall mean ± SEM for vehicle (white bars) and ibuprofen (black bars). Data analyzed by two-way ANOVA.

No apparent change in Sertoli cell number or topographical organization was observed in FEGA explants after exposure to 10^−5 ^M or 10^−4 ^M of ibuprofen, based on expression of AMH or cytokeratin 18 (KRT18) a marker of the immature Sertoli cell intermediate filaments, (Fig. 4A–F). This was further demonstrated by an unchanged Sertoli cell density after 72 h of exposure to 10^−5 ^M of ibuprofen (Fig. 4G).

**Figure 5:**
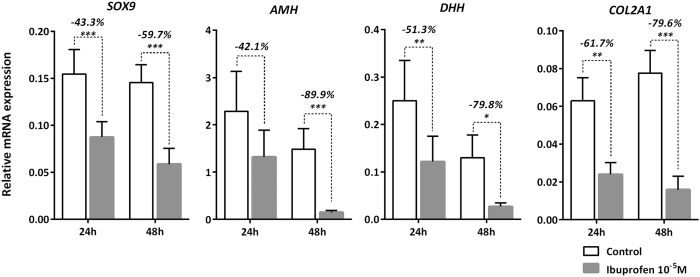
Ibuprofen and Sertoli cell markers. Quantitative RT-PCR for *SOX9, AMH, DHH* and *COL2A1* were performed on control testes (white bars) and testes treated with 10^−5 ^M of ibuprofen (grey bars) at 24 or 48 h of culture. Each column represents a pool of 11–15 fetal testes. Each bar shows the mean ± SEM of the fold change in target gene expression relative to the *RPLP0* reference gene. A non-parametric signed rank Wilcoxon test on paired data was performed (*p < 0.05; **p < 0.01; ***p < 0.001).

When the testes from the youngest human fetuses (7–7.9 GW) were exposed to 10^−5 ^M of ibuprofen, a significant decrease in AMH was seen from 48 h of culture onwards (−31.1% at 48 h (*p* < 0.05), −53.0% at 72 h (*p* < 0.0001), and −38.1% at 96 h (*p* < 0.05)) (Fig. 4H). When fetuses of 8–12 GWs were analyzed together, a dose-dependent suppression of AMH production was observed (slope β = −0.294, *p* < 0.0001), with −38.4% (*p* < 0.001) at 10^−5 ^M and −76.2% (*p* < 0.001) at 10^−4 ^M (Fig. 4I). The dose-dependent decrease of AMH levels was also seen both in the 8–9.9 GW group (slope β = −0.295, *p* < 0.0001), with −31.0% (*p* < 0.05) at 10^−7 ^M, −54.8% (*p* < 0.001) at 10^−5 ^M and −66.7% (*p* < 0.001) with 10^−4 ^M (Fig. 4J) and in the 10–12 GW group (slope β = −0.291, *p* = 0.003) (Fig. 4K). In accordance with these findings, the expression of *AMH* mRNA was found to be suppressed by ibuprofen exposure in hFEGA: −42.1% at 24 h and −89.9% (*p* < 0.001) at 48 h (Fig. 5). As with *AMH* mRNA, the mRNA of *SOX9*, a specific marker of the differentiation of Sertoli cells, was repressed after ibuprofen exposure: −43.3% (*p* < 0.001) at 24 h; and −59.7% (*p* < 0.001) at 48 h. The mRNA expression of the 2 *SOX9* targets investigated *DHH* and *COL2A1* was decreased by −51.3% (*p* < 0.01) and −61.7% (*p* < 0.01) at 24 h; and −79.8% (*p* < 0.001) and 79.6% (*p* < 0.001), respectively (Fig. 5).

We also investigated the effect of ibuprofen exposure on AMH production in the second trimester human fetal testis using the xenograft system. In contrast to the results in the first trimester testis there was no significant difference in AMH production in second trimester xenografts exposed to ibuprofen compared to vehicle-exposed controls (0.75 *versus* 1.34 ng/ml; *p* = 0.038; Fig. 4L).

### Ibuprofen alters germ cell markers

**Figure 6:**
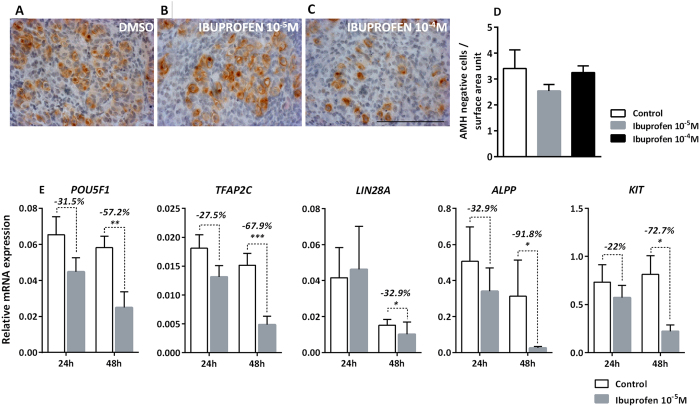
Ibuprofen and germ cells. (**A**–**C**) Representative images of M2A immunostaining in cultured explants of an 8.6-gestational week (GW) human fetal testis. M2A appears brown (3,3′-diaminobenzidine tetrahydrochloride staining), and sections were counterstained with hematoxylin. Scale bar = 100 μm. (**D**) Numbers of germ cells were determined by counting AMH-negative cells after culturing human fetal testis (*n* = 7 testes from 7 fetuses) in the presence of 0.01% of DMSO (Control, white bars) or 10^−5 ^M (grey bars) or 10^−4 ^M (black bars) of ibuprofen. Data are presented as the number of cells per surface area unit (0.01 mm^2^) (means ± SEM) based on 1 or 2 explants per treatment from 7 fetuses. A non-parametric signed rank Wilcoxon test on paired data was performed. (**E**) Quantitative RT-PCR for *POU5F1, TFAP2C, LIN28A, ALPP* and *KIT* was performed on control testes (white bars) and testes treated with 10^−5 ^M of ibuprofen (grey bars) at 24 or 48 h of culture. Each column shows a pool of 11 or 15 fetal testes. Each bar represents the mean ± SEM of the fold change in target gene expression relative to the *RPLP0* reference gene. A non-parametric signed rank Wilcoxon test on paired data was performed (*p < 0.05, **p < 0.01; ***p < 0.001).

Neither the morphology nor the density of the germ cells appeared altered after 72 h of exposure to 10^−5^ and 10^−4 ^M of ibuprofen (Fig. 6A–D). This contrasted with the expression of 5 germ cell genes which were repressed after 48 h of ibuprofen exposure: *POU5F1,* a transcription factor essential for the pluripotency maintenance in embryonic stem cells^43^, was significantly decreased by 57.2% (*p* < 0.01); *TFAP2C,* a factor believed to regulate the expression of several genes involved in cell growth and differentiation^44^, was also significantly decreased by 67.9% (*p* < 0.001); *LIN28A,* which regulates the germ cell pool^45^, was significantly decreased by 32.9% (*p* < 0.05); *ALPP* and *KIT*, which are typical markers of germ cells^46^,^47^, were significantly decreased by 91.8% (p < 0.05) and 72.7% (p < 0.05), respectively (Fig. 6E).

### Ibuprofen suppresses the production of prostaglandin PGE2, but not that of PGD2

**Figure 7:**
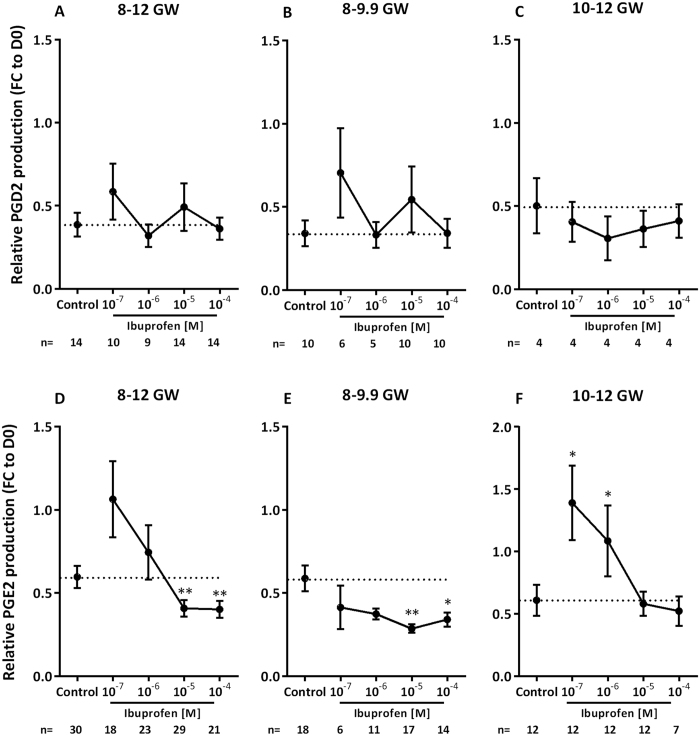
Effect of ibuprofen on human fetal testis PGD2 and PGE2 production. (**A**–**C**) PGD2 concentrations after 72 h of culture of 8 to 12 gestational week (GW) (**A**), 8–9.9 GW (**B**), and 10–12 GW (**C**) human fetal testes in the presence of the solvent DMSO (Control) or 10^−7^–10^−4^ M of ibuprofen. (**D**–**F**) PGE2 concentrations after 72 h of culture of 8–12 GW (**D**), 8–9.9 GW (**E**), and 10–12 GW (**F**) human fetal testes in the presence of DMSO (Control) or 10^−7^–10^−4^ M of ibuprofen. Results are expressed as fold change from the first day of culture (FC to D0) for both PGD2 and PGE2 productions. Data are means ± SEM (*n* = 4–10 testes from 4–10 fetuses for PGD2; *n* = 6−18 testes from 6–18 fetuses for PGE2). ANOVAs with a random embryo effect were performed using unstructured covariance matrices. A Wilcoxon test was performed for pairwise comparisons (*p < 0.05, **p < 0.01).

Cyclooxygenase enzymes (COX1 and COX2) are responsible for catalyzing the formation of prostaglandins from arachidonic acid, and ibuprofen is classically known to inhibit these enzymes^48^,^49^. We therefore assayed the prostaglandins PGD2 and PGE2, both previously found to be produced by the human fetal testis^26^. At all ages investigated, ibuprofen did not have a significant effect on PGD2 levels (Fig. 7A–C). In contrast, an ibuprofen-induced dose-dependent inhibition of PGE2 levels was demonstrated (slope β = −0.109, *p* = 0.03). Furthermore, significant inhibitory effects on PGE2 levels were observed in the 8–12 GW fetal testes after 72 h of exposure to 10^−5^ and 10^−4 ^M of ibuprofen (−31.6%, *p* < 0.01 at 10^−5 ^M; and −32.7%, *p* < 0.01 at 10^−4 ^M) (Fig. 7D). This ibuprofen-suppressive effect was more pronounced in the 8–9.9 GW fetal testes under the same conditions: −29.7% at 10^−7 ^M; −36.5% at 10^−6 ^M; −51.4% (*p* < 0.01) at 10^−5 ^M; and −42.0% (*p* < 0.05) at 10^−4 ^M (Fig. 7E), again with a significant dose-dependent suppression of PGE2 production (slope β = −0.122, *p* = 0.02). In the 10–12 GW testes, no significant inhibition of PGE2 was seen (Fig. 7F). However, high levels of PGE2 were observed when the 10–12 GW testes were exposed to the lowest doses of ibuprofen (+128.1% at 10^−7 ^M, *p* < 0.05 and +78.1% at 10^−6 ^M, *p* < 0.01).

## Discussion

The 3 most frequently consumed mild analgesics during the first trimester of pregnancy are paracetamol (also known as acetaminophen) and the NSAIDs aspirin and ibuprofen^1^. Several recent epidemiological studies have reported that the consumption of ibuprofen during the first trimester is associated with an increased risk of cryptorchidism and/or hypospadias^7^,^8^,^33^. In animals and humans, these congenital abnormalities are considered complex disorders involving either rare genetic traits, or abnormal endocrine activity or production in fetal testes^37^,^38^,^39^,^50^,^51^,^52^. Since the *in utero* assessment of ibuprofen’s effects on the development and function of human fetal testes is impossible for obvious ethical reasons, we developed an *ex vivo* model system which has already proven to be useful for studying the effects of paracetamol, NSAIDs (aspirin and indomethacin) and environmental chemicals (bisphenol A) on the human fetal testis during the first trimester^26^,^40^. In addition, as a complementary tool of investigation, we utilized a xenograft system which has been shown to model the effects of prolonged exposure to therapeutic doses of analgesics in the human testis during the second trimester^27^.

The present study demonstrates, through the use of the FEGA, that concentrations which are equivalent to or even lower than peak plasma levels of ibuprofen^53^,^54^ markedly affect the biology of the 2 major human fetal testicular somatic cell populations, as well as of the germ cells, and that these effects occur during specific periods of human fetal testis development. This occurred without any obvious changes in the number of these cells or in the general morphology of the testis at any age. Thus, ibuprofen markedly decreased testosterone levels and steroidogenesis at large. This inhibitory effect was observed using both the immunoassay of testosterone and GC/MS-MS analysis, and was found to be dose-, time- and age-dependent. Of note is that this appeared restricted to the 8–9.9 GW fetal testes. Androgens play an essential role in the masculinization of the urogenital tract^55^. This narrow window of ibuprofen sensitivity corresponding to the younger fetal ages investigated is similar to that previously observed for aspirin and indomethacin^26^. This likely reflects the fact that Leydig cells go through markedly different steps of differentiation and thus biological states^56^, some of which appear to be resistant to ibuprofen’s anti-androgenic effects. Indeed, we were able to demonstrate that testosterone production was not reduced by exposure to ibuprofen in late first trimester human fetal testis (10–12 GW) in the *ex vivo* culture system, nor in second trimester human fetal testis (14–17 GW) using the xenograft approach. The ibuprofen-induced testosterone inhibition during the specific 8–99 GW time window contrasts greatly with the effects observed in previous studies in aspirin- and indomethacin-treated fetal testes *ex vivo*. In fact, we found that the latter NSAIDs actually stimulate human testis testosterone levels^26^, rather than suppressing them as in the case of ibuprofen. Another striking difference is that, while paracetamol was previously found to reduce both plasma levels of testosterone and the weight of androgen-dependent seminal vesicles in the xenograft model-system^27^, ibuprofen had no effect in the xenograft-system model as revealed here. These differences are likely to reflect the different nature and thus mechanisms of action of the two analgesics considered, ibuprofen being a NSAID while paracetamol is not.

The present work also reveals that at least 3 steps of steroidogenesis are significantly affected *ex vivo* by ibuprofen, as it suppressed the transcripts encoding CYP11A1, CYP17A1, and HSD17B3. CYP11A1 is important as it catalyzes the first reaction of steroidogenesis (*i.e.* the conversion of cholesterol to pregnenolone). CYP17A1 catalyzes 17α*-*hydroxylation of progesterone and pregnenolone, and also converts 17α-hydroxypregnenolone to DHEA and 17α-hydroxyprogesterone to androstenedione. CYP17A1 is typically regarded as a key determinant of testosterone production, susceptible to both androgen production regulation and perturbation^57^,^58^. HSD17B3 catalyzes the ultimate conversion of androstenedione to testosterone, androstenedione being the first androgen produced in the downstream chain line of steroidogenesis. The present study represents the first GC/MS-MS global analysis of fetal testicular steroidogenesis demonstrating that, in addition to suppression of testosterone, the effect of ibuprofen on the expression of CYP17A1 also negatively impacted androstenedione and to a greater extent 5α-DHT. Further supporting evidence for ibuprofen being an anti-androgenic compound is the observation that low doses of this NSAID (5–6 mg/kg/day for 35 days) inhibited plasma levels of testosterone in adult male mice^59^. Ibuprofen has also been shown to inhibit the human UDP-glucuronosyltransferases UGT2B15 and UGT2B17, and the latter is very important for glucuronidation and testosterone excretion^60^.

It is important to note that not only androgens were impacted by ibuprofen but also INSL3, which is the peptidic hormone produced by Leydig cells and which was dose-dependently inhibited by ibuprofen. In accordance with this, *INSL3* expression levels were also suppressed by ibuprofen. *Insl3 k*nock-out mice are cryptorchid^50^,^61^ and some mutations of the human *INSL3* gene have been associated with cases of cryptorchidism^38^. The first phase of testicular descent which occurs in humans between 8 and 17 GW is controlled by INSL3^28^,^38^,^51^,^62^. In a previous study, 8–12 GW human fetal testes treated with paracetamol had reduced INSL3 production^26^, and in the present study ibuprofen exposure in testis explants at the same gestational age for 72 h induced significant alterations in INSL3 production. These findings support the hypothesis that ibuprofen-induced suppression of INSL3 expression and production could be related to the increased risk of cryptorchidism in boys whose mothers were exposed to ibuprofen during pregnancy^7^.

Interestingly, ibuprofen also induced a dose-dependent suppression of AMH production in the human fetal testis *ex vivo*. To the best of our knowledge, ibuprofen is the first endocrine disruptor which is found to display direct suppressive action on the endocrine function of the human (and other mammalian) fetal Sertoli cells. However, the reduced AMH production following ibuprofen exposure was restricted to the first trimester testis during a specific period from 7–9.9 GW. Exposure in late first trimester (10–12 GW) explants and in second trimester (14–17 GW) xenografts did not result in a significant change in AMH production although the variability between samples clearly increased in the late first trimester. In addition, there was a large variability in AMH production between fetuses for the second trimester xenografts which was accounted for using two-factor analysis as previously described^27^. The difference observed here between the FEGA *ex vivo (i.e.* decreased levels of AMH) and the xenograft model system (*i.e.* no change in AMH levels) could result from the age-difference of the fetal testes used; it also cannot be excluded, however, that the intrinsic difference of the two assay-systems discussed before^28^ could also account for it. Taken together, the results obtained *ex vivo* indicate that there exist specific ‘windows of sensitivity’ during which certain Sertoli cell function may be affected by exposure to ibuprofen. It is likely that the ibuprofen suppression of *SOX9* mRNA expression evidenced in this study along with several of its known targets (*AMH, DHH, COL2A1*), represents the basis by which ibuprofen also suppresses AMH.

Our study reveals not only that ibuprofen disturbs the endocrine homeostasis of the testis, in both the Sertoli and Leydig cells, but also that exposure to this NSAID markedly alters the expression of the germ cells genes *POU5F1, TFAP2C, LIN28A, ALPP*, and *KIT*. These genes are expressed in a population of mitotic fetal germ cells named gonocytes. NSAIDs have been shown to promote cell cycle arrest and apoptosis thereby decreasing the initiation and/or progression of various cancers (colorectal, bladder, skin, esophageal adenocarcinoma, ovarian)^63^,^64^,^65^,^66^. Whether ibuprofen effects occur by similar mechanisms remains to be established. In addition, whether ibuprofen-induced alteration of germ cell gene expression results from a direct effect of this NSAID on the human gonocytes, or if it is secondary to the marked suppression of the endocrine function of both Sertoli and Leydig cells is also unknown.

PGD2 and PGE2, the 2 prostaglandins investigated in this study, are thought to be involved in the differentiation of the fetal testis. In fact, exposing mouse testis explants (11, 12, and 14 days post-coitum) to PGE2 prevented the differentiation of testis cords^67^. Furthermore, mice deficient in *Ptgds,* which encodes the lipocalin-type prostaglandin D2 synthase enzyme responsible for PGD2 synthesis, display unilateral cryptorchidism^68^. However, here, we show that exposure to ibuprofen had no significant effect on PGD2 production. In contrast, ibuprofen induced marked changes in PGE2 levels. It is interesting to note that, as in our previous study on paracetamol, aspirin, and indomethacin, human fetal testis PGD2 production appears to be much less sensitive to the effects of ibuprofen than PGE2^26^. The precise mechanism of action underlying the inhibitory effect of ibuprofen on PGE2 production remains to be explored. PGE2 is known to modulate immune and inflammatory responses through its receptors EP1 to EP4^69^, and EP2 has recently been detected within the rat fetal testis^70^. It also remains to be determined what the consequences of ibuprofen-induced suppression of PGE2 might be on the human fetal testis biology and development. It would be important to understand which testicular cell type(s) is/are responsible for PGE2 production and which ones express the receptor for PGE2 within the human fetal testis.

Of all the analgesics that we have tested so far using our *ex vivo* human fetal testis system^26^, ibuprofen appears to be the agent which alters the human fetal endocrine balance with the widest range of effects as it affects all the major testicular cell types. Interestingly, paracetamol, aspirin, indomethacin, and ibuprofen each display their own endocrine-disrupting signatures (Table 1). The only unequivocally shared effect observed with these 4 analgesics is their inhibitory action on prostaglandin E2 production in 8–9.9 GW fetuses. However, whereas paracetamol and aspirin did not have any effect on testosterone production at this stage of gestation, the same group of testes treated with ibuprofen had lower testosterone production. Several other differences between analgesic effects also exist: ibuprofen - unlike aspirin but similar to paracetamol - suppresses INSL3 production. Furthermore, aspirin increased AMH production whereas paracetamol had no such effect, and ibuprofen showed a dose-response inhibition of this Sertoli cell produced hormone (Table 1). Taken together, these observations indicate that each drug has its own mechanism of action within the human fetal testis. Due to the different analgesic-induced biological signatures induced within the fetal testis, it is likely that this explains why when pregnant women simultaneously use more than one analgesic (paracetamol, aspirin, and ibuprofen), the risk of cryptorchidism observed is almost 10 times higher (odds ratio 2 fold versus 17 fold) than when a single painkiller is used^7^. Likewise, simultaneous use of both paracetamol and NSAIDs was found to be associated with significantly shorter AGD in boys, while exposure to paracetamol only was not significantly associated with shorter AGD^24^. In any case, the different therapeutic indications and contra-indications of these medications, *i.e.* paracetamol and of the NSAIDs, also reflect their different structures and properties. The present data complement our previous findings, which show that analgesics can behave as endocrine disruptors during key stages of the development of the urogenital tract^26^. This study provides new mechanistic explanations for the increased risk of cryptorchidism and hypospadias observed after *in utero* exposure to these medications by different groups.

In conclusion we show that exposure to therapeutic levels of ibuprofen during specific ‘windows of sensitivity’ can result in multiple effects on Sertoli-, Leydig- and germ-cell development and function in the human fetal testis. This includes effects on production of several testicular hormones during the first trimester. These findings are based on the results of 2 different model systems of human fetal testis development. Whilst they cannot be directly translated into recommendations for the use of ibuprofen in humans they provide experimental support to the epidemiological association between analgesic use and the development of male reproductive disorders, evidence that would support the avoidance of ibuprofen use during the first trimester where practicable.

## Materials and Methods

### Ethics statement

First trimester (7–12 gestational week) and second trimester (14–17 gestational week) human fetuses were obtained from abortions performed at the Rennes Sud Hospital in Rennes, France and the Royal Infirmary of Edinburgh, Edinburgh, UK, respectively. None of the pregnancy terminations were due to fetal abnormalities. Women gave written informed consent as per the legal procedures put in place by the French national biomedical research agency (authorization #PFS09-011; Agence de la Biomédecine) and the Declaration of Helsinki – Ethical Principles for Medical Research Involving Human Subjects. All experiments were performed in accordance with relevant guidelines and regulations. Ethical approval for the study was obtained from Rennes Sud Hospital local ethics committee (notice #11-48) and South East Scotland Research Ethics Committee (reference number: LREC08/S1101/1).

### Human fetal testis collection

First-trimester human fetal testes were recovered from the abortion aspiration products using a binocular microscope (Olympus SZX7, Lille, France). They were immediately placed in ice-cold phosphate-buffered saline (PBS) solution. Second trimester human fetal testes were recovered and placed into ice-cold ‘xenograft media’ containing Liebowitz L-15 with glutamine, 10% fetal bovine serum, 1% penicillin/streptomycin and 1% non-essential amino acids (all Sigma, Poole, UK).

### Culture and xenograft procedures

The testes were cut into explants of less than 1 mm^3^ according to a standardized protocol previously described^26^,^40^. For the testes younger than 8 GW, 2 wells were designed to accommodate 1 testis and either control or 10^−5 ^M of ibuprofen treatment. These testes were immediately exposed to treatments. For the 8 to 10 GW group, 4 wells were prepared for 4 different culture conditions (1 control and 3 ibuprofen concentrations). These wells were each half a testis in length and contained 2 to 3 pieces of a single testis. For the older testes (10 to 12 GW), 6 wells for 6 different culture conditions (1 control and 5 ibuprofen concentrations) were created, each containing 1 or 2 testis pieces from a single testis. The explants were cultured in inserts (0.4 μm pores; Falcon, Becton-Dickinson, Le Pont de Claix, France) placed in 24-well companion culture plates (Becton-Dickinson). Human chorionic gonadotrophin (Sigma Aldrich, Saint-Quentin, France) was added at a concentration of 0.1 IU/mL^71^, and the cultures were incubated at 37 °C for 96 h in a humidified atmosphere of 95% air and 5% CO2. The medium was removed every 24 h and divided into at least 2 aliquots that were immediately snap-frozen on dry ice and stored at −80 °C. To assess dose-response effects, after the first 24 h of culture (D0) the explants were exposed to either the control, DMSO at a final concentration of 0.1%, or to the ibuprofen treatment, with concentrations of 10^−7^ M to 10^−4^ M added to the medium.

For xenograft studies, human fetal testes were grafted into castrate host mice as previously described^27^. Briefly, small pieces (1 mm^3^ approx; 4–6 per mouse) of testis tissue were placed subcutaneously, either side of the midline, under the dorsal skin of the mice using a 13 G cancer implant needle (Popper and Sons, US). In general, 3–6 mice were xenografted with tissue from each fetus, and mice were maintained for 7 days to ensure vascularisation before any host treatments commenced. One week after grafting, host mice commenced treatment with subcutaneous injection of human chorionic gonadotropin (20 IU hCG every 72 hours; Pregnyl, Organon Laboratories) to mimic the human *in utero* environment^72^. Host mice were also randomly allocated to receive a therapeutic regimen of either ibuprofen (10 mg/kg three times daily), or vehicle (corn oil) by daily oral administration with analysis 1 hour after the final dose. Host mice were sacrificed by cervical dislocation, and blood was obtained by cardiac puncture for assessment of plasma testosterone, AMH and ibuprofen. Testosterone production and action was assessed by measuring plasma testosterone and seminal vesicle weight. No differences were observed between vehicle- and ibuprofen-exposed host mice in terms of body weight, number of grafts retrieved or total graft weight (Fig. S1).

### Immunostaining and stereology

Immunohistochemistry was performed on 4% paraformaldehyde-PBS and Bouin solution-fixed, paraffin-embedded tissues, as previously described^26^. The Sertoli cells were labeled with an AMH goat primary antibody (1:100; Santa Cruz Biotechnology, CA, USA). Leydig cells were stained with a rabbit anti-cytochrome P450, family 11, subfamily A, polypeptide 1 (CYP11A1) antibody (1:250; Sigma Aldrich). A mouse M2A primary antibody (1:100; Abcam, Paris, France) was used for gonocyte immunolabeling. For the AMH and M2A antibodies, antigens were retrieved for 40 min at 80 °C in 10 mM citrate buffer, pH 6.

A NanoZoomer 2.0-RS scanner (Hamamatsu, Tokyo, Japan) was used to capture pictures of the whole slides at 40x magnification. The surface area of 5 to 10 sections randomly selected within the whole explant were calculated with NDP.view software (Hamamatsu). ImageJ software (US National Institutes of Health, Bethesda, MD, USA) was used to perform the stereological cell counting. Germ and Sertoli cells were identified and counted as intra-cordal AMH-negative and AMH-positive cells, respectively.

### Hormone assays

Testosterone was assayed using a specific radioimmunoassay (RIA): a direct testosterone RIA with an intra-assay coefficient of variation (CV) ≤ 8.6% and an inter-assay CV of 11.9% (Immunotech, Beckman Coulter, Villepinte, France). A specific RIA was also used for insulin-like 3: a human INSL3/RLF RIA kit with intra- and inter-assay CVs of ≤15 and 7%, respectively (Phoenix Pharmaceuticals, Strasbourg, France). AMH levels were assayed using an AMH/MIS Enzyme-Linked Immunosorbent Assay (ELISA) kit with an intra-assay CV of 3.2 to 12.3% and inter-assay CV of 5.8 to 14.2% (Immunotech, Marseille, France). PGD2 was measured with ELISA using a prostaglandin D2-MOX EIA Kit having an intra-assay CV of 8 to 15% and an inter-assay CV of 10 to 17% (Cayman Chemical, Ann Arbor, MI, USA). PGE2 was also assayed using a prostaglandin E2 EIA Kit (intra-assay CV 3.7 to 30.4% and inter-assay CV 6.4 to 35%) (Cayman Chemical).

The hormone assay data from the culture system using the testes of the fetuses younger than 8 GW were expressed as hormone production fold change as compared to the control testis. Since the older testes were cut into explants, the first 24 hours of culture (day 0 without treatment, or D0) served as the baseline for normalization of hormone production in each testicular sample after 24, 48, and 72 h of exposure to DMSO or ibuprofen. Depending on fetal age, we established cut-offs for normal relative testosterone production in the control experiments, calculated at D3 normalized to the D0 result in the same culture well. These were >0.7 for the 8–9.8 GW explants, and ≥0.1 for the 10–12 ones.

### Global analysis of steroids

The procedures for applied sample preparation and GC-MS/MS measurements have been previously described^73^,^74^. Tissue samples were homogenized after addition of 50:50 v/v methanol and water (300 μl/10 mg tissue). Prior to extraction for quantification according to isotopic dilution, tissue homogenates, and cell culture media (200 μl) were spiked with internal standards (17β-estradiol-16,16,17-d3; 5α-androstan-3α,17β-diol-16,16,17-d3; etiocholanolone-2,2,3β,4,4-d5; 17β-testosterone-16,16,17-d3; 4-androsten-3,17-dione-19,19,19-d3; 5α-androstan-17β-ol-3-one-16,16,17-d3; and 17-methyltestosterone-d3). Non-polar compounds were extracted from the aqueous phase (pH 5.2) with diethyl ether, followed by a liquid/liquid partitioning to separate androgens and estrogens. Both fractions were purified on 200 mg Upti-Clean SI-S cartridges (Interchim, Montluçon, France), then derivatized with a silylation reagent as described by Courant *et al*.^74^. Two micro-liters of each final extract were injected into the GC-MS/MS system. The detection and quantification of the targeted compounds were performed on a spectrometer (Bruker, Fremont, CA, USA). Electron impact (70 eV) was the ionization mode used for androgens, while negative chemical ionization (80 μA filament current) with methane reactant gas was applied for estrogens. The source temperature was set to 250 °C and the transfer line to 300 °C. The chromatographic conditions were previously described by Courant *et al*.^74^. The mass spectrometer was operated in multiple reaction monitoring (MRM) acquisition mode, and argon was used as collision gas at 2.0 mTorr. The diagnostic signals were monitored for the targeted compounds have been reported elsewhere^73^,^74^.

For xenografts, testosterone and ibuprofen were extracted from mouse plasma by solid phase extraction using 10 mg HLB Oasis cartridges (Waters, UK). Briefly, 50 μL plasma was enriched with 1 ng 13C3-Testosterone (QMX, UK) as internal standard. Cartridges were conditioned with methanol and water, the sample loaded and washed with water, 5% methanol and then eluted with 1 mL methanol. The eluate was reduced to dryness under nitrogen at 40 °C and reconstituted in mobile phase (70 μL water/acetonitrile (70:30, v/v)). Chromatographic separation was achieved by injecting 30 μL sample onto an Acquity UPLC system with an ACE Excel C18-AR column (150 × 2.1 mm; 2 μm) protected by a Kinetex KrudKatcher (Phenomenex) and operated at 30 °C. The mobile phase consisted of 0.1% formic acid (Sigma Aldrich) in water and 0.1% formic acid in acetonitrile (B) at a flow rate of 0.5 mL/min. Gradient elution from 30–100% B was achieved, with a total run time of 9 mins. Testosterone, and Ibuprofen eluted at 5.40 and 5.7 min respectively. Following separation, testosterone and ibuprofen were detected on a QTrap 5500 triple quadrupole mass spectrometer (Sciex, Warrington, UK) operated in positive ion electrospray mode (5.5 kV, 550 °C, ion source gas 60/40). Transitions monitored were m/z 289.1 → 97.0, and *m/z* 156.1 → 114.1 for testosterone and ibuprofen, respectively. The ratio of the peak area of each analyte to the internal standard was used to generate a calibration curve and using linear regression analysis the amount of each analyte was calculated.

### RNA extraction and quantitative Polymerase Chain Reaction (qPCR)

RNA extractions from testes were done using a NucleoSpin XS kit (Macherey-Nagel, Hoerdt, France) according to the manufacturer’s instructions. Total RNA (250 ng) was reverse transcribed with random primers and Moloney murine leukemia virus reverse transcriptase (Invitrogen, ThermoFisher, Courtaboeuf, France). Quantitative PCR was performed according to the manufacturer’s instructions using the SYBR Green master mix (Applied Biosystems, ThermoFisher) with a 0.5 μl cDNA template in an Applied Biosystems 7500 Real-Time PCR system. The amplification program was as follows: a 2 min holding stage at 50 °C; initial denaturation of 10 min at 95 °C; 40 cycles of 15 sec denaturation at 95 °C; and 1 min at 60 °C for annealing and extension. Dissociation curves were produced using a thermal melting profile performed after the last PCR cycle. To avoid amplification of contaminating genomic DNA, primer pairs were selected on either side of an intron. *RPLP0* and *RPS20* mRNA were used as internal controls for normalization^75^ (Table 2). Results were calculated by the ΔΔC_T_ method as n-fold differences in target gene expression with respect to the reference gene and the calibration sample (made of an equal mix of each of the samples tested).

### Statistical analysis

Hormone measurements (means ± SEM) are expressed either as percentages of the values as compared to the control testes (7 to 7.9 GW fetuses), as the fold change from the respective untreated first day of culture (D0; 8–12 GW fetuses), or as absolute values for serum in xenografted mice.

Testosterone and AMH production as a function of culture duration in 7–7.9 GW testes were analyzed using repeated measures analysis of variance (ANOVA) on ranks. Ibuprofen dose-response relationships were assessed for testosterone, INSL3, AMH, PGD2, and PGE2 using variance analyses, with the treatment as explanatory variable and a random fetus effect. To normalize the data, logarithm transformations were used for INSL3, AMH, PGD2 and PGE2 data. The dose was transformed into a categorical variable (ranging from 1 for the lowest dose to 4 for the highest one) then introduced into the models as continuous variables. The corresponding slope β therefore represents the change in hormone secretion for each supplementary dose unit. Pairwise differences between the ibuprofen treatments and controls were analyzed using the non-parametric Wilcoxon signed-rank test. Quantitative PCR data, GC-MS/MS data, and cell-counts were also analyzed with the Wilcoxon test. For analysis of AMH, testosterone and ibuprofen in xenografts, a two-way ANOVA was performed. Significance was defined as a confidence level of *p* < 0.05. Statistical analyses were performed using SAS software (SAS/STAT version 9.3; SAS Institute Inc., Cary, NC) and SigmaPlot 12.0 software (Systat Software, San Jose, CA, USA).

## Additional Information

**How to cite this article:** Ben Maamar, M. *et al*. Ibuprofen results in alterations of human fetal testis development. *Sci. Rep.* **7**, 44184; doi: 10.1038/srep44184 (2017).

**Publisher's note:** Springer Nature remains neutral with regard to jurisdictional claims in published maps and institutional affiliations.

## Supplementary Material

Supplementary Figure 1

## Figures and Tables

**Table 1:** Summary of the endocrine-disrupting signatures of each tested analgesic *ex vivo.*

Cell type	Hormone	Paracetamol	Ibuprofen	Aspirin	Indomethacin
Leydig cell	Testosterone	⇔	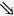 1	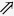	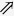
	INSL3	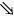	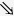	⇔	⇔
Sertoli cell	AMH	⇔	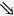	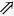	⇔
Unknown	PGD2	⇔	⇔	⇔	⇔
	PGE2	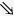	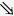 or 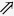 2	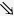	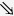

Data for paracetamol, aspirin and indomethacin come from^26^.

^1^After 24, 48 and 72 h of exposure.

^2^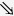 In the 8–9.9 GW and 8–12 GW testes at 10^−5^ and 10^−4 ^M; 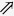 in the 10–12 GW testes at 10^−7^ and 10^−6 ^M.

**Table 2:** Primers used for qPCR.

Gene	Upstream primer	Downstream primer	Product length (bp)	Annealing temp °C
*ALPP*	TCTGGGTACTCAGGGTCTGG	ATCGCTACGCAGCTCATCTC	101	62
*AMH*	CGCCTGGTGGTCCTACAC	GAACCTCAGCGAGGGTGTT	162	67
*BZRP*	GGCTTCACAGAGAAGGCTGT	ACTGACCAGCAGGAGATCCA	87	66
*COL2A1*	GGCAATAGCAGGTTCACGTACA	CGATAACAGTCTTGCCCCACTT	57	53
*CYP11A1*	AGACCTGGAAGGACCATGTG	TCCTCGAAGGACATCTTGCT	435	65
*CYP17A1*	GTGGAGACCACCACCTCTGT	GCTGAAACCCACATTCTGGT	108	67
*DHH*	TGATGACCGAGCGTTGTAAG	GCCAGCAACCCATACTTGTT	196	67
*HSD3B2*	GCCTGTTGGTGGAAGAGAAG	GCAGGCTCTTTTCAGGAATG	158	62
*HSD17B3*	TCCTTGGCCTCTCTACTCCA	AGACAGCATATGGGGTCAGC	125	62
*INSL3*	CCCAGAGATGCGTGAGAAGT	CCAGCCACTGTAGCAACTCA	229	68
*KIT*	TTCTTACCAGGTGGCAAAGG	AAATGCTTTCAGGTGCCATC	209	62
*LIN28A*	AAGCTGCACATGGAAGGGTT	CCGCCTCTCACTCCCAATAC	138	67
*POU5F1*	TACTCCTCGGTCCCTTTCC	CAAAAACCCTGGCACAAACT	131	67
*RPLP0*	TCTACAACCCTGAAGTGCTTGAT	CAATCTGCAGACAGACACTGG	167	66
*RPS20*	AACAAGCCGCAACGTAAAA	ACGATCCCACGTCTTAGAA	96	67
*SOX9*	AACGCCTTCATGGTGTGG	TCTCGCTCTCGTTCAGAAGTC	124	59
*SRD5A3*	AGGAATGCCTACATAACAGGGAA	CTCCAAATGGGATCCTGTGGT	181	62
*STAR*	GGCTGGCATGGCCACAGACT	TTGGGCAGCCACCCCTTGA	162	77
*TFAP2C*	CCGGTCCTTGCGGGAGAAGTT	CTGGTTTACTAGGAAATTCGGCTTCACA	164	75
