# Supplementary material for: Ibuprofen results in alterations of human fetal testis development
Source: Sci Rep. 2017 Mar 10;7:44184. doi: 10.1038/srep44184 (PMC5345102; doi:10.1038/srep44184)
Supplement: Supplementary Figure 1 [file srep44184-s1.pdf]

Ibuprofen results in alterations of human fetal testis development

Millissia Ben Maamar[1], Laurianne Lesné[1], Kristin Hennig[2], Christèle Desdoits-Lethimonier[1], Karen R. Kilcoyne[3], Isabelle Coiffec[1], Antoine D. Rolland[1], Cécile Chevrier[1], David M. Kristensen[4], Vincent Lavoué[5], Jean-Philippe Antignac[2], Bruno Le Bizec[2], Nathalie Dejucq-Rainsford[1], Rod T. Mitchell[3], Séverine Mazaud-Guittot[1], Bernard Jégou[1,6*]

[1]Institut national de la santé et de la recherche médicale (Inserm), Institut de recherche en santé, environnement et travail (Irset – Inserm UMR 1085), 9 Avenue Léon Bernard, F-35000 RENNES, France. Université de Rennes 1, F-35043 RENNES, France.

[2]LUNAM Université, Oniris, USC INRA 1329, Laboratoire d'Etude des Résidus et Contaminants dans les Aliments (LABERCA), Nantes, F-44307, France.

[3]MRC Centre for Reproductive Health, University of Edinburgh, Queens Medical Research Institute, 47 Little France Crescent, Edinburgh, EH16 4TJ.

[4]Laboratorium of Genomic and Molecular Biomedicine, Department of Biology, University of Copenhagen, Ole Maaløes Vej 5, DK-2200 Copenhagen N, Denmark.

[5]CHU de Rennes, Service de Gynécologie, Hôpital Sud, 16, boulevard de Bulgarie, F-35700 Rennes, France; Université de Rennes 1, Faculté de Médecine, F-35043 RENNES, France.

[6]Ecole des hautes études en santé publique (EHESP), Avenue Léon Bernard, F-35043 RENNES, France.

***To whom correspondence should be addressed:** bernard.jegou@inserm.fr

Figure S1.

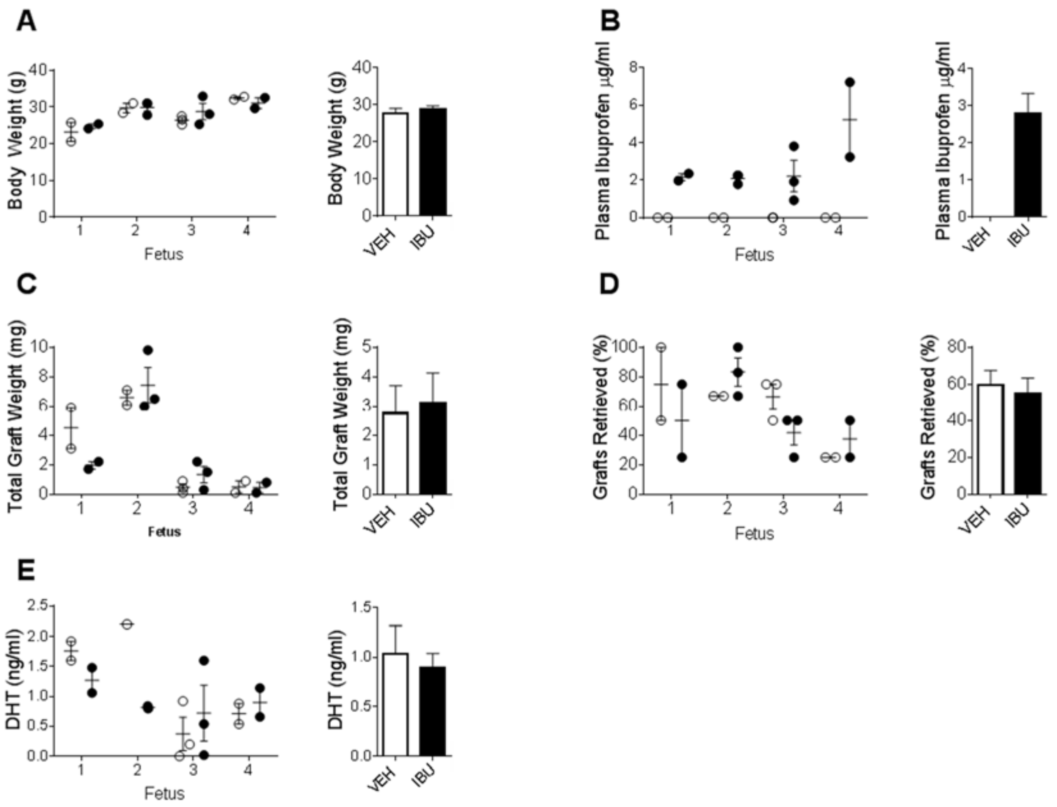

Figure S1. Body weight (A), Grafts retrieved (B) and Total graft weight (C) for individual host mice carrying human fetal testis xenografts (14-17 GW; n=4) exposed for 7d to vehicle (corn oil; open circles) or ibuprofen (10 mg/kg 3 times daily; closed circles); with overall mean ± SEM for vehicle (white bars) and ibuprofen (black bars). (D) Plasma ibuprofen concentration in vehicle (open circles) and ibuprofen (closed circles) exposed host mice. Data analyzed by two-way ANOVA.
